# Evaluation of a nurse-led chronic kidney disease clinic: a single-centre cohort study

**DOI:** 10.1093/ckj/sfag084

**Published:** 2026-03-16

**Authors:** Hannah Wallace, Qiumian Wang, Kylie Simoni, Min Jun, Sandra Crikis, Craig Nelson

**Affiliations:** Faculty of Medicine, Dentistry and Health Science, The University of Melbourne, Melbourne, VIC, Australia; Western Health Chronic Disease Alliance, Western Health, Melbourne, VIC, Australia; The George Institute for Global Health, UNSW Sydney, Sydney, NSW, Australia; Western Health Chronic Disease Alliance, Western Health, Melbourne, VIC, Australia; Western Health Chronic Disease Alliance, Western Health, Melbourne, VIC, Australia; The George Institute for Global Health, UNSW Sydney, Sydney, NSW, Australia; Faculty of Medicine and Health, UNSW Sydney, Sydney, NSW, Australia; Western Health Chronic Disease Alliance, Western Health, Melbourne, VIC, Australia; Faculty of Medicine, Dentistry and Health Science, The University of Melbourne, Melbourne, VIC, Australia; Western Health Chronic Disease Alliance, Western Health, Melbourne, VIC, Australia

**Keywords:** chronic kidney disease, models of care, nurse practitioner, quality-improvement

## Abstract

**Background:**

The objective of this study was to evaluate the clinical outcomes and patient satisfaction of a new nurse practitioner chronic kidney disease (CKD) clinic.

**Methods:**

This was a prospective cohort study of all patients who had their first appointment with the early CKD nurse practitioner from October 2023 to October 2024, with data extracted on 1 April 2025. The outcomes were assessed across three domains: (domain 1) change in clinical outcomes of kidney function, albuminuria and blood pressure, (domain 2) prescription of guidelines concordant medications and (domain 3) patient satisfaction with care as assessed by a survey.

**Results:**

There were 95 patients with a median age of 63 years [interquartile interval (IQI) 51–76], and 45.3% were female. Cardio-metabolic comorbidity was common; 82.8% of patients had hypertension, 55.6% diabetes and 25.2% cardiovascular disease. At referral the median urine albumin–creatinine ratio was 32.3 mg/mmol (IQI 11.2–53.5) and this was lower at discharge, 12.4 mg/mmol (IQI 2.9–25.3, *P *< .001). Systolic and diastolic blood pressure were lower at discharge [mean difference mmHg (95% confidence interval, *P*-value): 10.1 (6.58–13.54, <.001) and 5.2 (2.6–7.7, <.001), respectively]. There was no change in median estimated glomerular filtration rate. More patients at discharge were on a renin–angiotensin system inhibitor (88.4% versus 76.8%), sodium-glucose cotransporter 2 inhibitor (53.6% versus 28.4%), mineralocorticoid receptor antagonist (23.1% versus 5.2%) and glucagon-like peptide-1 receptor agonist (23.1% versus 9.6%). The survey response rate was 51.6% with patients reporting high satisfaction with the service.

**Conclusion:**

The CKD nurse practitioner clinic was effective at implementing guideline-directed medical therapies for early CKD and associated with improvements in blood pressure and albuminuria.

## Graphical Abstract

**Graphical Abstract:**
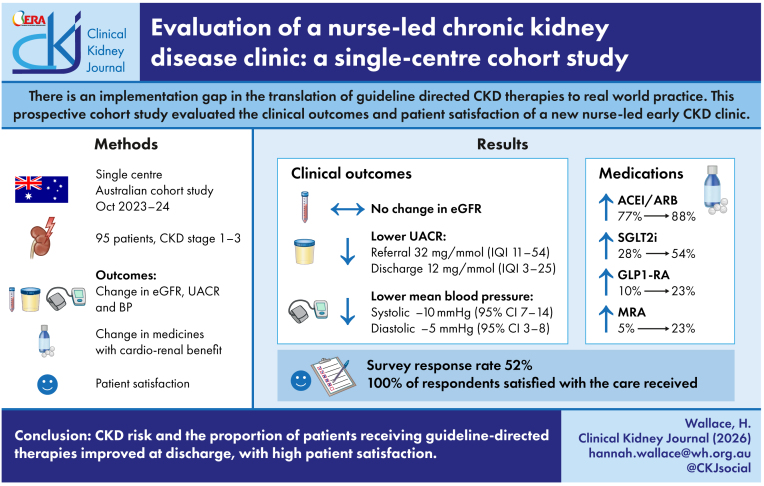

KEY LEARNING POINTS **What was known:**

- There is an implementation gap in the translation of guideline-directed therapies to real world practice.
- Expanded nursing roles have been utilized as part of a multidisciplinary care team to improve uptake of guideline-directed care.

**This study adds:**

- In this single-centre cohort study, nurse practitioner–led clinical care was effective at establishing guideline-directed care for patients with chronic kidney disease in the context of cardio-metabolic risk factors.
- Patients seen in the nurse practitioner clinic had a reduction in their risk of kidney disease progression.

**Potential impact:**

- Expanded nursing roles have the potential to improve the implementation gap by increasing access to specialist services and early implementation of guideline-directed kidney care.
- Further research is required to understand the longer-term impacts of this model of care.

## INTRODUCTION

Chronic kidney disease (CKD) is estimated to affect up to 14% of the Australian population [1], with the most common cause of end-stage renal failure in Australia attributable to diabetes [2]. There is good evidence that the implementation of guideline-directed medical therapies can reduce and even prevent progression of CKD and reduce cardiovascular risk, in addition to lifestyle changes and risk-factor control. For the benefits to be realized, these need to be implemented into routine clinical care. Internationally, an implementation gap between best practice CKD care and real-world practice has been well established [3–6]. Similarly, Australian primary care data have highlighted priority areas in the testing and management of patients with CKD, with gaps in albuminuria testing and uptake of guideline-directed medical therapies such as angiotensin-converting enzyme (ACE) inhibitors and/or angiotensin-receptor blockers (ARB), and sodium-glucose cotransporter 2 (SGLT2) inhibitors [7–9].

The high and growing prevalence of CKD means nephrology physician care is focused on the treatment of patients with advanced CKD and those with glomerular or genetic diseases [10, 11]. Patients with earlier disease are primarily cared for in primary care [11, 12] or have a long wait time to be seen in government-funded nephrology services. New models of care are required to address both workforce shortages and the implementation gap. One such model proposed has been expanded nursing roles [13, 14], whereby nurse practitioners undergo additional training in a specific field of practice enabling them to work both independently and collaboratively in providing patient care, including prescribing of relevant medications [15]. The addition of nurse practitioners in CKD shared care arrangements has found improvements in the proportion of patients on guideline-directed therapy [16–18], though the results were mixed in relation to kidney endpoints [13]. In Australia, a nurse practitioner model of care has been shown to be effective at improving testing and management within an Indigenous community [19], and more broadly CKD nurse practitioner models have been associated with good patient satisfaction with care [20]. In this quality improvement research, we aimed to establish whether a nurse practitioner model of care was effective in the prescription of guideline-directed medical therapy and improving clinical outcomes of patients with early-stage CKD (stage 1–3) referred to nephrology services, and whether patients were satisfied with the quality of care they received.

## MATERIALS AND METHODS

### Early CKD nurse practitioner clinic

The early CKD nurse practitioner clinic was established at an Australian metropolitan tertiary service in an area with a known high prevalence of CKD and renal replacement therapy [21], and diabetes [22]. Referrals to the nephrology outpatient service with an estimated glomerular filtration rate (eGFR) ≥30 mL/min/1.73 m^2^ and urine albumin–creatinine ratio (UACR) ≤150 mg/mmol with CKD likely due to diabetes and/or hypertension (as based on referral information) were triaged to the nurse practitioner clinic. The clinic aimed to review patients one to three times prior to discharge back to the primary care physician with a comprehensive management plan, or referral to the main nephrology clinics for ongoing care if required. The initial review was conducted in person, with subsequent reviews conducted either in person or telehealth depending on patient preference and clinical need. Clinical cases were discussed at a weekly multidisciplinary meeting with the nurse practitioner and a nephrologist. Australia has a universal healthcare system. Medications were prescribed in line with evidence-based guidelines [10] and availability on the Australian Pharmaceutical Benefits Scheme (PBS) [23]. The PBS is a national government program that subsidizes essential medication costs to all Australians, with greater subsidization for concession card holders. Patients were discharged to primary care once established on guideline-directed care if their Kidney Failure Risk Equation was <3%–5% at 5 years and if no further work up was required for CKD due to a suspected non-cardio-metabolic cause.

### Study design

This was prospective cohort study of all patients who had their first appointment with the CKD nurse practitioner from 24 October 2023 to 23 October 2024, with data extracted on 1 April 2025. Patients who failed to attend subsequent appointments or had not completed follow-up were excluded (Supplementary data, Fig. S1). Patients were offered the opportunity to provide anonymous survey feedback after discharge from the CKD nurse practitioner clinic. The survey was adapted from patient satisfaction with nurse practitioner care for presenting problem from The Nurse Practitioner Research Toolkit by Gardner *et al*. [24]. The survey was in English and participants from non-English speaking backgrounds were able to have a family member to assist them. Study data were collected and managed using REDCap electronic data capture tools hosted at Western Health [25, 26].

### Outcomes

The aim of this analysis was to establish whether the CKD nurse practitioner clinic was effective at initiating guideline-recommended therapy, improving clinical outcomes and acceptable to patients. The outcomes were assessed across three domains: (domain 1) change in clinical outcomes of eGFR, UACR and blood pressure, (domain 2) prescription of guidelines concordant medications and (domain 3) patient satisfaction with care. Clinical outcomes were compared between referral and discharge appointment with the CKD nurse practitioner. It was hypothesized that there would be no change in eGFR (domain 1) during the short follow-up period with the potential for decline with initiation of medicine known to cause an acute decline in eGFR [27, 28]. Medication recommendations were based on the Kidney Disease: Improving Global Outcomes guideline [10] and included ACE inhibitor or ARB, SGLT2 inhibitor, mineralocorticoid receptor antagonist (MRA) and, in patients with type 2 diabetes, glucagon-like peptide-1 receptor agonists (GLP-1RA). Patient satisfaction with care was assessed using a survey (Supplementary data, Appendix S1) including basic demographics and medical conditions, knowledge of kidney disease prior to review, communication and overall evaluation.

### Statistical analysis

Descriptive statistics were used to describe the cohort at baseline. Normally distributed data is reported with a mean and standard deviation (SD) and non-normally distributed as a median and interquartile interval (IQI). Paired *t*-tests were used to compare baseline and discharge clinical outcomes for normally distributed data. The Shapiro–Wilk test was used to check the assumption of normality. For non-normally distributed data the Wilcoxon signed rank test was used. For domain 2, the percentage of patients on therapy at baseline compared with discharged was calculated, and the McNemar’s test used to determine statistically significant differences. A subgroup analysis of patients with UACR ≥22.6 mg/mmol was conducted for ACE inhibitor and/or ARB, SGLT2 inhibitor and MRA as the Australian subsidized medication listing for SGLT2 inhibitors for a CKD indication and non-steroidal MRA in patients with diabetes and CKD require a UACR of ≥22.6 mg/mmol. Survey responses were analysed using descriptive statistics, with a thematic analysis of free text responses. All statistical analyses were performed with Stata version 17.0 (Stata, College Station, TX, USA).

### Ethics

This study was approved by University of Melbourne Human Research Committee (STEMM 2) 2023-27529-44500-3 and Western Health Office for Research LNR/100956/WH-2023-386207.

## RESULTS

### Baseline characteristics

There were 95 patients seen in the CKD nurse practitioner clinic, with a median age of 63 years (IQI 51–76), and 45.3% were female (Table 1). One-third of patients had a primary language other than English. The median Charlson Comorbidity Index was 3 (IQI 1–6) and cardio-metabolic comorbidity was common, with 82.8% of patients having a history of hypertension, 55.6% diabetes and 25.2% established cardiovascular disease. Risk of CKD progression was moderately increased in 16.8%, high in 50.5% and very high in 31.5% of patients (Supplementary data, Table S1). Patients were seen within 6 weeks of referral and seen over a median of 3 visits (IQI 2–4) with a median of 112 days (IQI 62–204) in the service.

**Table 1:** Baseline characteristics of cohort.

	Overall cohort number (%)	Survey respondents number (%)^^a^^
Total	95	49
Female	43 (45.3)	21 (42.8)
Age, years (median, IQI)	63 (51–76)	
Age category, years		
18–49	22 (23.2)	10 (20.8)
50–69	36 (37.9)	20 (41.6)
≥70	37 (38.9)	18 (37.5)
Non-English-speaking background	32 (33.7)	14 (28.5)
Referred by GP	88 (92.6)	
eGFR (mL/min/1.73 m^2^)		
≥90	21 (22.1)	
60–89	25 (26.3)	
45–59	18 (18.9)	
30–44	29 (30.5)	
15–29	2 (2.1)	
Albuminuria (mg/mmol)		
Normal (< 3)	13 (13.7)	
Moderately increased (≥3–30)	31 (32.6)	
Severely increased (>30)	51 (53.7)	
Charlson Comorbidity Index (median, IQI)	3 (1–6)	
Comorbidities		
Diabetes	55 (55.6)	27 (57.4)
Cardiovascular disease	24 (25.2)	11 (23.4)
Hypertension	82 (82.8)	39 (83.0)
Medication number	6 (3–9)	5 (3–7)

Data are presented as *n* (%) unless otherwise specified.

a Self-reported. GP, general practitioner.

### Clinical outcomes

At baseline, median eGFR was 58 mL/min/1.73 m^2^ (IQI 42–84) with no change at discharge (60.5 mL/min/1.73 m^2^, IQI 42–84, *P *= .815) (Table 2). Referral median UACR was 32.3 mg/mmol (IQI 11.2–53.5), and was significantly lower at discharge (12.4 mg/mmol, IQI 2.9–25.3, *P *< .001). Systolic and diastolic blood pressure were lower at discharge [mean difference mmHg (95% confidence interval, *P*-value): 10.1 (6.58–13.54, <.001) and 5.2 (2.6–7.7, <.001), respectively].

**Table 2:** Renal function, albuminuria and blood pressure at baseline and discharge from a CKD nurse practitioner clinic.

	Baseline	Follow-up	Difference	*P*-value
eGFR, median (IQI)	58.0 (42.0–84.0)	60.5 (42.0–84.0)	0 (IQI –4 to 4)	.8151
UACR, median (IQI)	32.3 (11.2–53.5)	12.4 (2.9–25.3)	15.6 (IQI 2.4–32.4)	<.001
Blood pressure systolic, mean (SD)	132.1 (19.1)	122.1 (12.1)	10.1 (95% CI 6.58–13.54)	<.001
Blood pressure diastolic, mean (SD)	76.2 (13.2)	71.2 (10.7)	5.17 (95% CI 2.61–7.74)	<.001

CI, confidence interval.

**Figure 1:**
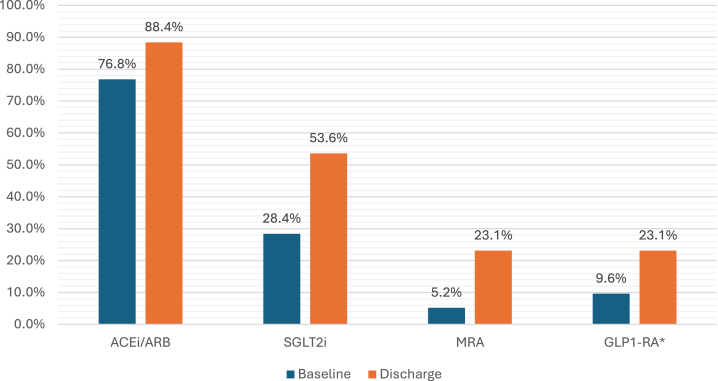
Proportion of patients receiving guideline-directed therapy at baseline and discharge.

The proportion of patients on guideline-directed therapy was higher at discharge across all medication classes (Fig. 1). At referral 76.8% of patients were on an ACE inhibitor or ARB compared with 88.4% at discharge (*P *= .017). At referral 28.4% of patients were on an SGLT2 inhibitor, with 53.6% being discharged on an SGLT2 inhibitor (*P *< .001). In patients not on an SGLT2 inhibitor at discharge, 90% did not meet the criteria (at time of discharge) for the government medication subsidiary for either a diabetes indication or a CKD indication. The remainder had another reason not to commence or for future review. At baseline, MRA were prescribed in 5.2% of patients and at discharge 23.1% (*P *< .001). In patients with diabetes, 9.6% were on a GLP-1RA at baseline, and this increased to 23.1% at discharge (*P *< .001). A subgroup analysis of patients with an elevated UACR ≥22.6 mg/mmol at baseline found a similar increase in use of guideline-directed therapies (Supplementary data, Table S2).

Over three-quarters of patients were able to be discharged to their primary care physician (77.8%). The remainder were referred to the standard nephrology clinic for ongoing follow-up. Reason for ongoing nephrology care was due to ongoing high risk of CKD progression (≥3%–5% risk 5-year kidney failure risk) or concern for an alternate pathology, with eight cases having microhaematuria and a concern for potential immunoglobulin A nephropathy and three patients requiring further work-up for multiple myeloma or monoclonal gammopathy of renal significance.

### Patient satisfaction

**Figure 2:**
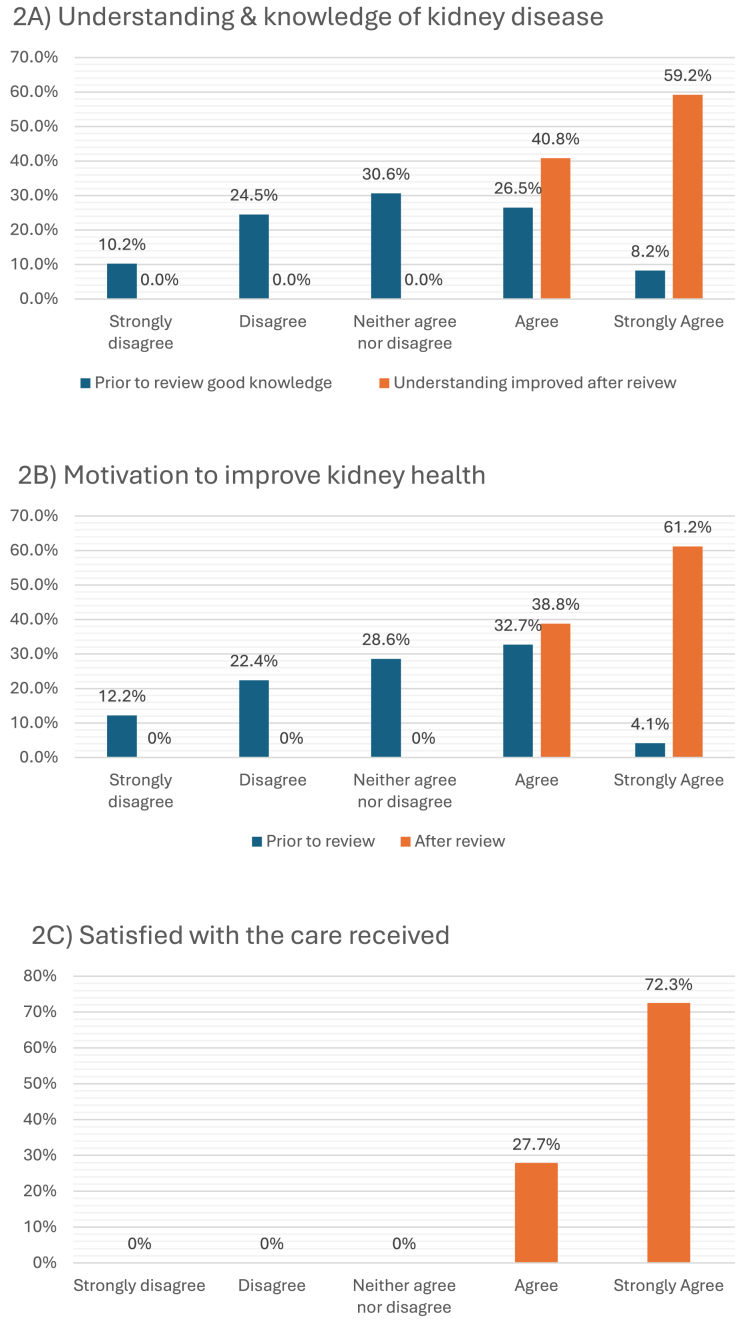
Survey responses regarding CKD nurse practitioner clinic on understanding of kidney disease pre with improvement post (A), motivation to improve kidney health pre and post (B), and satisfaction if care received (C).

The survey response rate was 51.6%, with 45.3% being female and 28.6% speaking a primary language other than English. There was high patient satisfaction, with 100% of respondents agreeing or strongly agreeing that they were satisfied with the care they received, that their understanding of kidney disease was better after seeing the CKD nurse practitioner and that they were motivated to improve their kidney health (Fig. 2). Additionally, 95.6% reported that the kidney nurse recommended changes in their lifestyle, with 81.6% reporting that they definitely would make the changes recommended and 14.3% agreeing somewhat. Nearly half of patients provided a free-text response of feedback of their experience. Key themes raised were that patients felt comfortable, received patient-centred care, there was good communication both with them and their primary care practitioner, and they felt the CKD nurse practitioner was very knowledgeable (Table 3).

**Table 3:** Themes from free text survey response regarding feedback of experience with the kidney nurse.

Key themes	Supporting quotes
Comfortable	‘felt very comfortable, she helped me relax’
	‘I felt really comfortable talking to her’
	‘was very reassuring’
	‘she’s comfortable to talk to’
	‘she makes you feel very comfortable to ask any questions’
	‘she was patient with my questions and most of all made me feel comfortable’
Received patient-centred care	‘Your outstanding commitment to patient care is truly commendable’
	‘The nurse I saw provided exceptional care… Having the opportunity to discuss things at length helped me understand my kidney health and how to improve it more effectively and really improved my motivation to be more proactive about my health’
	‘kindness and thoughtfulness through good communication and attainable health advice during the entire visit’
Communication	‘She took the time to explain things clearly’
	‘prompt communication’
	‘good communication’
	‘awesome communication’
	‘her communication with my GP made everything easier’
	‘best thing is that she was working with my GP to get better results and lifestyle changes’
Knowledgeable	‘Greater access to knowledgeable specialist care was also appreciated’
	‘well knowledge[d] in my condition’
	‘nurse was very knowledgeable’
	‘is very knowledgeable and thorough’
	‘informative’

GP, general practitioner.

## DISCUSSION

In this cohort study of patients attending a newly established early CKD nurse practitioner clinic we found that patients were appropriately established on guideline-directed medical therapies with improvement in albuminuria and blood pressure parameters. Importantly, patients were highly satisfied with the clinical care they received. These findings suggest that an early CKD nurse practitioner clinic model of care is effective in improving the implementation of guideline-directed therapies in patients referred to nephrology outpatient services.

Prior literature supports the addition of a nurse practitioner to the care team, with key studies reporting improved blood pressure control and uptake of cardio-renal protective medications [16, 29, 30]. Furthermore, James *et al*. [18] found that patients cared for by CKD nurse practitioners as an alternative to nephrology care were more likely to be on ACE inhibitors and/or ARBs compared with those cared for by primary care physician or nephrologist, with no difference in clinical outcomes including kidney failure and all-cause mortality when compared with nephrology care. Our findings support that an early CKD nurse practitioner–led model of care is effective in initiating guideline-directed therapies and improving UACR and blood pressure control, thereby reducing risk of CKD progression. This model of care enables timely review of patients with less advanced stages of CKD who would normally be triaged to a non-urgent physician review (recommended review within 365 days) and therefore more time on therapy with cardio-renal benefits. Patients in whom further investigation for potential glomerular disease was required were also appropriately identified through the service.

A strength of this clinical model is the opportunity to provide in-depth CKD education and lifestyle counselling through motivational interviewing. Lifestyle change and risk-factor management is widely accepted as essential in the management of cardio-kidney-metabolic syndrome [31]. To be done effectively, techniques such as motivation interviewing are often used. Motivational interviewing requires building of rapport and adequate time to understand a patient’s readiness for change, potential barriers and goal setting [32]. Appointments with the CKD nurse practitioner were longer than a standard outpatient consults to allow for this (60 min first review with subsequent reviews 30 min, compared with 45 and 20 min for physician appointments). The survey feedback indicated the majority of patients felt that they would be able to implement the changes recommended and were motivated to improve their kidney health.

This study has some important limitations to consider. This study was evaluating a new clinical service and was not randomized nor was there a control group, and as such we are unable to determine how it compared with standard of care with nephrology physician review. Secondly, the clinical follow-up period concluded when patients were discharge from the service, so it is unknown what proportion of patients referred to ongoing primary care management were continued on therapy. Whilst the survey feedback indicated high patient motivation for change it was also unknown to what extent patients implemented and continued lifestyle changes. Future research is required to determine the longer-term outcomes of this intervention in relation to renal outcomes, ongoing medication use and sustainability of lifestyle changes. Additionally, an economic analysis to understand potential cost savings of this model of care would be important.

## CONCLUSION

The CKD nurse practitioner clinic is an effective model of care to increase the number of patients on guideline-directed medical therapies for early CKD and was associated with significant improvements in blood pressure control and albuminuria reduction. Future research is required to understand the longer-term outcomes and potential cost savings.

## Supplementary Material

sfag084_Supplemental_Files

## Data Availability

The data underlying this article are available in the article and in its online supplementary material.
