# Supplementary material for: Evaluation of a nurse-led chronic kidney disease clinic: a single-centre cohort study
Source: Clin Kidney J. 2026 Mar 16;19(4):sfag084. doi: 10.1093/ckj/sfag084 (PMC13153467; doi:10.1093/ckj/sfag084)
Supplement: sfag084_Supplemental_Files [file sfag084_Supplemental_Files.zip › Supplemental Appendix Survey_CKDNPClinic.pdf]

Survey

Record ID ______________________

Background Information

What is your gender?
- ◯ Male
- ◯ Female
- ◯ Another term

What is your age?
- ◯ aged 18- 49
- ◯ aged 50-69
- ◯ aged 70 and over

Is English the primary language spoken at home?
- ◯ Yes
- ◯ No

What the primary language spoken at home? ______________________

What is your employment status?
- ◯ Employed full time
- ◯ Employed part time
- ◯ Unemployed
- ◯ Disability pension
- ◯ Aged pension
- ◯ Student
- ◯ Home duties
- ◯ Prefer not to answer

Kidney appointment and referral

How long did you have to wait for the kidney nurse today?
- ◯ Less than 15 minutes
- ◯ Less than 30 minutes
- ◯ 30-60 minutes
- ◯ More than 60 minutes

Did you have an interpreter for you appointment today?
- ◯ Not required
- ◯ Not avaliable
- ◯ Yes

How did you attend your kidney nurse appointments?
- ◯ In person
- ◯ Telehealth
- ◯ Phone
- ◯ Home review
- ◯ Mixture of in person and telehealth/phone

How many appointments have you had with the kidney nurse?
- ◯ 1
- ◯ 2
- ◯ 3
- ◯ More than 3

Do you know why you were referred to the kidney nurse?
- ◯ Yes
- ◯ No

How many times have you seen your general practitioner (GP or family doctor) in the last 12 months?	◯ Once<br>◯ 2-3 times<br>◯ 4-5 times<br>◯ 6 or more times<br>◯ I do not have a regular GP
How many times have you been admitted to hospital in the last 12 months?	◯ 0<br>◯ 1 time<br>◯ 2-3 times<br>◯ 4-5 times<br>◯ 6 or more times
How many chronic (long term) medical problems do you have?<br>(for example blood pressure, diabetes, heart disease, lung disease etc).	◯ 0<br>◯ 1<br>◯ 2-3<br>◯ 4-5<br>◯ 6 or more
What medical problems do you have?<br><br>Please tick all that apply	☐ Kidney disease<br>☐ Blood pressure (or on medications for blood pressure)<br>☐ Diabetes<br>☐ Heart disease<br>☐ Liver disease<br>☐ Stroke or mini stroke<br>☐ Chronic obstructive lung disease (also called emphysema or smoking lung disease)
How many medications do you take?	◯ 0<br>◯ 1<br>◯ 2<br>◯ 3<br>◯ 4<br>◯ 5<br>◯ 6<br>◯ 7<br>◯ 8<br>◯ 9<br>◯ 10 or more
If you selected 10 or more how many medications do you take?	______________________

Kidney knowledge

Think back to before your first kidney appointment with the kidney nurse and answer these questions.

	Strongly disagree	Disagree	Neither agree nor disagree	Agree	Strongly agree
Before seeing the kidney nurse, I believe I had a good knowledge of kidney disease	◯	◯	◯	◯	◯

	No definitely not	No I don't think so	Yes somewhat	Yes definitely	Not applicable
Before seeing the kidney nurse, I already knew how to improve my kidney health	○	○	○	○	○
Before seeing the kidney nurse, I already felt motivated to improve my kidney health	○	○	○	○	○

During appointments (think about all the appointments you had with the kidney nurse)

	No definitely not	No I don't think so	Yes somewhat	Yes definitely	Not applicable
Did the kidney nurse discuss information about kidney disease with you?	○	○	○	○	○
Did you understand the information the kidney nurse discussed?	○	○	○	○	○
Did the Kidney nurse recommend any changes in your lifestyle or habits that would improve your kidney health or prevent illness?	○	○	○	○	○
Did the kidney nurse help you create a plan to change your habits or lifestyle?	○	○	○	○	○
Do you think you will make the changes the kidney nurse suggested?	○	○	○	○	○

Which of the following did the kidney nurse discuss with you?
Including all your appointments with the kidney nurse.
Please tick all that apply.

- ☐ Smoking cessation
- ☐ Weight management / weight loss
- ☐ Dietary advice
- ☐ Exercise

Did the Kidney nurse prescribe any medications?

- ○ Yes
- ○ No

Did the kidney nurse discuss any medications you were already taking with you?

- ○ Yes
- ○ No

	No definitely not	No I do not think so	Yes somewhat	Yes definitely	Not applicable
Did you receive enough information about these medications?	○	○	○	○	○
Did you understand the discussion about your medications?	○	○	○	○	○

Nurse Communication

	Never	Almost never	Sometimes	Usually	Almost always	Always
How often did the kidney nurse explain things in a way that was easy to understand?	○	○	○	○	○	○
How often did the kidney nurse listen carefully to you?	○	○	○	○	○	○
How often did the kidney nurse spend enough time with you?	○	○	○	○	○	○
How often did the kidney nurse seem to know all the important information about your medical history?	○	○	○	○	○	○
How often did the kidney nurse give you clear instructions about how to manage your kidney health?	○	○	○	○	○	○
How often do you feel you could talk to the kidney nurse about anything, even things that you might not tell anyone else?	○	○	○	○	○	○

Overall Evaluation

	Strongly disagree	Disagree	Neither agree nor disagree	Agree	Strongly agree
My understanding of kidney disease was better after seeing the kidney nurse.	○	○	○	○	○
The kidney nurse helped me understand how I can improve my kidney health.	○	○	○	○	○
I feel motivated to improving my kidney health.	○	○	○	○	○
My understanding of my kidney medicines improved after seeing the kidney nurse.	○	○	○	○	○
I was satisfied with the care I received from the kidney nurse.	○	○	○	○	○

Did you have any further feedback about your experience with the kidney nurse?

____
