# Supplementary material for: Evaluation of a nurse-led chronic kidney disease clinic: a single-centre cohort study
Source: Clin Kidney J. 2026 Mar 16;19(4):sfag084. doi: 10.1093/ckj/sfag084 (PMC13153467; doi:10.1093/ckj/sfag084)
Supplement: sfag084_Supplemental_Files [file sfag084_Supplemental_Files.zip › Supplementary Appendix 10Sept25.docx]

**Supplementary** **Appendix:**

Supplementary Appendix 1: Patient Survey

**Supplemental Appendix Figures:**

Supplementary Figure 1: Study flow chart

**Supplemental Appendix Tables:**

Supplementary Table 1: CKD heat map

Supplementary Table 2: Prescription medications at baseline and discharge overall, and by sub-group with a UACR >22.6 mg/mmol at baseline.

**Supplementary** **Figure 1:** Study flow chart


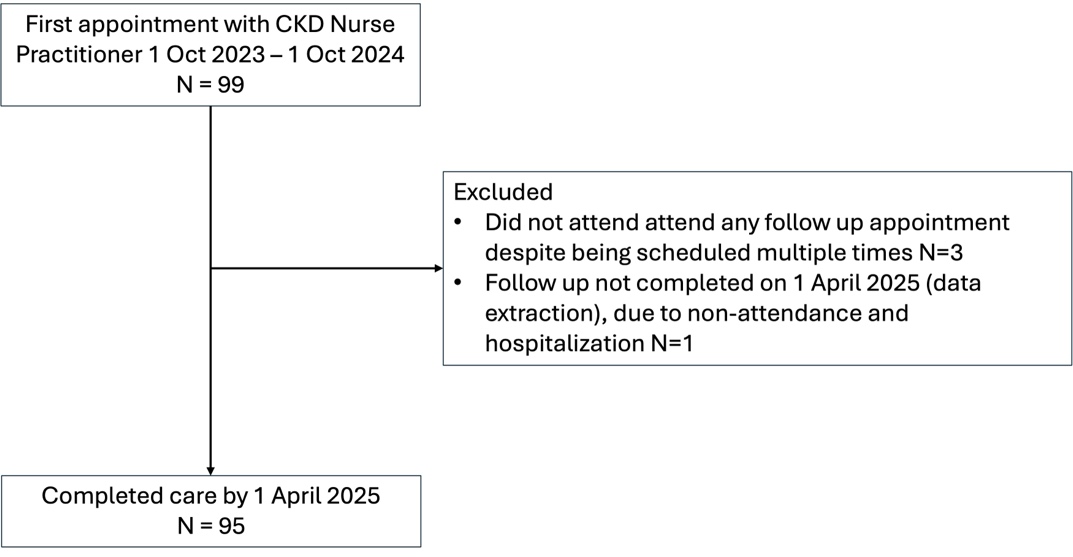


**Supplementary** **Table 1:** CKD heat map

|  |  | Albuminuria categories, UACR mg/mmol | | |
| --- | --- | --- | --- | --- |
|  |  | A1 normal to mildly increased  <3 mg/mmol | A2 Moderately increased  3-30 mg/mmol | A3  Severely increased  >30 mg/mmol |
| eGFR category  mL/min/1.73m2 | G1  > 90 | - | 4 (4.2) | 17 (17.9) |
|  | G2  60-89 | 1 (1.01) | 8 (8.4) | 16 (16.8) |
|  | G3a  45-59 | 4 (4.2) | 8 (8.4) | 6 (6.3) |
|  | G3b  30-44 | 7 (7.4) | 10 (10.5) | 12 (12.6) |
|  | G4  15-29 | 1 (1.01) | 1 (1.01) | - |

**Supplementary** **Table 2:** Prescription medications at baseline and discharge overall, and by sub-group with a UACR >22.6 mg/mmol at baseline.

| **Medications** | **Baseline**  Number (%) | **Follow up**  Number (%) | **p-value** |
| --- | --- | --- | --- |
| **Overall** |  |  |  |
| ACEI/ARB | 73 (76.8) | 84 (88.4) | 0.016 |
| SGLT2i | 27 (28.4) | 51 (53.6) | <0.001 |
| MRA | 5 (5.2) | 22 (23.1) | <0.001 |
| GLP1RA* | 5 (9.6) | 12 (23.1) | 0.016 |
| **Baseline UACR > 22.6 mg/mmol** |  |  |  |
| ACEI/ARB | 50 (79.4) | 63 (100.0) | <0.001 |
| SGLT2i | 20 (31.8) | 42 (66.7) | <0.001 |
| MRA | 1 (1.6) | 17 (27.0) | <0.001 |
